# Pathophysiological analysis of idiopathic sudden sensorineural hearing loss by magnetic resonance imaging: A mini scoping review

**DOI:** 10.3389/fneur.2023.1193104

**Published:** 2023-04-20

**Authors:** Michihiko Sone, Masumi Kobayashi, Tadao Yoshida, Shinji Naganawa

**Affiliations:** ^1^Department of Otorhinolaryngology, Nagoya University Graduate School of Medicine, Nagoya, Japan; ^2^Department of Radiology, Nagoya University Graduate School of Medicine, Nagoya, Japan

**Keywords:** idiopathic sudden sensorineural hearing loss, MRI, imaging, high signal, blood-labyrinth barrier, endolymphatic hydrops

## Abstract

**Objective:**

To summarize the pathophysiological analysis of idiopathic sudden sensorineural hearing loss (ISSNHL) by magnetic resonance imaging (MRI), focusing on the findings of high signal or endolymphatic hydrops (EH) in the inner ear.

**Methods:**

We summarize the published studies of our research group regarding the pathophysiological analysis of ISSNHL on MRI and review related clinical articles that have reported significantly high signal or the existence of EH in ears with ISSNHL.

**Results:**

Pre-contrast high signal on MRI may indicate minor hemorrhage or increased permeability of surrounding vessels to the perilymph, whereas post-contrast high signal indicates breakdown of the blood–labyrinth barrier, in which irreversible changes would lead to poor prognosis. In some cases of ISSNHL, primary EH could be pre-existing and may be a risk factor for the onset of ISSNHL.

**Conclusion:**

Analysis of ISSNHL by cutting-edge MRI evaluation could provide useful information for elucidating its pathophysiology and for predicting prognosis in this disease.

## Introduction

The inner ear is difficult to investigate because it is surrounded by the otic capsule. In addition to experimental investigations using animal models, temporal bone analysis has generally been performed to elucidate the pathology of inner ear diseases. Computed tomography (CT) provides useful information regarding middle ear diseases but is inadequate for investigation of inner ear diseases such as idiopathic sudden sensorineural hearing loss (ISSNHL), a representative inner ear disease that causes sudden onset of SNHL with unknown etiology. Although several forms of pathogenesis have been hypothesized, such as viral infection or vascular compromise, as of yet none have been confirmed.

Magnetic resonance imaging (MRI) was first applied mainly to detect cerebellopontine angle tumors in patients with acute or fluctuating hearing loss. According to recent clinical practice guidelines for sudden hearing loss, MRI evaluation is recommended for evaluating retrocochlear pathology (1). Several studies have reported a correlation between labyrinthine enhancement on post-contrast MRI and the clinical findings in patients with hearing loss (2, 3).

Three-dimensional (3D) fluid-attenuated inversion recovery (FLAIR) can demonstrate hemorrhage and high protein concentration, both of which are difficult to detect on T1- and T2-weighted MRI. Moreover, compared with two-dimensional (2D) FLAIR, 3D FLAIR has the advantages of significantly less prominent flow artifact from cerebrospinal fluid and better depiction of structures in the cistern (4), and is thus suitable for the evaluation of inner ear disturbances. Technological advances and the development of 3D FLAIR have enabled the elucidation of the pathology of inner ear diseases in more detail.

Our group was the first to report high signal in the inner ear on 3D-FLAIR in patients with ISSNHL (5), and we have evaluated various other inner ear disorders using this technique. In patients with Meniere’s disease (MD), our group first demonstrated the presence of endolymphatic hydrops (EH) on MRI performed 24 h after intratympanic injection of a gadolinium-based contrast agent (GBCA) (6). Further technological advances enabled visualization of EH on MRI performed 4 h after intravenous injection of a GBCA (7, 8). Evaluation of EH on MRI in this manner has led to breakthroughs in identifying the pathophysiology of other inner ear disorders, including ISSNHL, as well as in the diagnosis of MD.

In the present paper, we describe the proposed pathophysiology of ISSNHL using cutting-edge MRI evaluation, relate our experiences, and review related clinical articles focusing on high signal and endolymphatic hydrops (EH) in the inner ear.

## Materials and methods

We summarize the published studies of our research group that specifically investigated the pathophysiological process of ISSNHL, focusing on the existence of high signal or EH in inner ears with this disease. We also reviewed related clinical literature published in English-language journals from 2006 to the present, which were identified by searching electronic databases (PubMed, Web of Science, ScienceDirect, and Scopes). Here we discuss papers that have reported significant findings regarding the proposed pathophysiology of ISSNHL based on cutting-edge MRI evaluation.

## Results

### High signal in the inner ear with ISSNHL

In 2006, Sugiura and our colleagues were the first to report high signal on 3D-FLAIR in the inner ears of patients with ISSNHL (5). They demonstrated that half of the patients with this disease had high signal in the affected ear on pre-contrast 3D-FLAIR at 3 T, and speculated that this finding might indicate minor hemorrhage, increased concentration of protein that had passed through blood vessels due to increased permeability, or disruption of cells in the inner ear. Enhancement following administration of a GBCA suggests breakdown of the blood–labyrinth barrier (BLB). In 2008, another paper from our department reported pre-contrast high signal in the cochleae of affected ears on 3D-FLAIR, which suggested a poor hearing prognosis in ISSNHL (9). Post-contrast high signal considered the result of breakdown of the BLB would indicate increased permeability due to inflammation in the inner ear (9). Thereafter, we performed a semiquantitative evaluation based on the property of contrast of cochlear fluid, as signal intensity ratio (SIR) between the cochlea and cerebellum in patients with ISSNHL, and proposed SIR as a good indicator of disruption of the BLB (10). Berrettini et al. demonstrated that 3D FLAIR was positively associated with pretreatment hearing loss and presence of vertigo, and described the value of MRI-based indicators such as mild hemorrhage, acute inflammation, or breakdown of the BLB for elucidating pathologic conditions in inner ears with ISSNHL (11). A comprehensive meta-analysis of the correlation between 3D FLAIR findings and outcome variables of ISSNHL found that the degree of hearing loss was more severe in ears with high signal and that the hearing recovery rate was significantly less in ears with high signal than in those without high signal (12). Affirmative findings for ISSNHL on 3D FLAIR have been reported subsequently (13); however, methodological differences in terms of the methods of MRI assessment, contrast-medium administration protocol, and assessment criteria for hearing improvement might have led to inconsistencies in the results (14). Song et al. (15) have also reported that correlations between abnormal enhancement and clinical prognosis varied among investigations.

3D FLAIR is highly sensitive for detecting asymmetric cochlear signal abnormality in ears with ISSNHL (16); however, the accuracy of evaluation depends on the degree of imaging sensitivity for detecting abnormal signal in the target organ. Signal characteristics of post-contrast 3D-FLAIR MRI may be related to the interval between disease onset and the time of the MRI examination (17). Naganawa and our colleagues have introduced heavily T_2_-weighted 3D-FLAIR (hT_2_W-3D-FLAIR), which is more sensitive to subtle T_1_-changes in fluid compared to regular contrast-enhanced 3D-FLAIR (18, 19). Our investigation of ears with ISSNHL demonstrated higher sensitivity to signal alterations in diseased cochleae on hT_2_W-3D-FLAIR than on regular 3D-FLAIR (20). We investigated signal intensity in inner ears with ISSNHL in a quantitative evaluation of hT_2_W-3D-FLAIR images, with the aim of identifying lesion-specific disturbances and their prognoses (21). The results showed that high post-contrast signal intensity in the apical–middle turns, but not in the basal turns, was related to worse hearing recovery at low-tone frequencies in patients with severe hearing loss, indicating irreversible changes with breakdown of the BLB in the apical–middle turns. Conversely, low post-contrast signal intensity in the apical–middle turns was related to better hearing recovery at low-tone frequencies, indicating reversible changes in the apical–middle turns. Increased permeability at the BLB may be lower in ears with ISSNHL than in those with MD (22); however, irrespective of the presence of disease, our study revealed a difference in vascular permeability at the BLB between older and younger patients (greater vascular permeability of the BLB in older patients) (23).

### EH in inner ears with ISSNHL

The condition of endolymphatic hydrops is characterized by distention of the space containing endolymph into the normally perilymph-occupied space. EH can occur in the cochlear duct, sacculus, utricle, and semicircular canals. MD is a representative type of EH. We first demonstrated the existence of EH in patients with MD in the clinical setting in 2007, by visualizing EH on MRI performed 24 h after intratympanic injection of a GBCA diluted eightfold with saline into the middle ear cavity (6). Since that time, technological advances have enabled visualization of EH on MRI performed 4 h after intravenous injection of GBCA (7, 8). Evaluation of EH on MRI has been a breakthrough in the diagnosis of MD, including assessment of the relationships between the clinical and physiological findings and the degree of EH. Moreover, MRI evaluation has revealed that EH exists extensively not only in ears with typical MD, but also in those with various other otological disorders. Numerous studies have reported the significance of EH on MRI and recommended MRI for verifying inner ear pathology in patients with SNHL, vertigo, and tinnitus and also for improving the management of EH-related diseases (24).

Our group conducted the first trial that attempted to visualize EH among cases of ISSNHL, however, there was no obvious existence of EH in the inner ears (10). Other clinical entities of sudden onset sensorineural hearing loss include acute low-tone sensorineural hearing loss (ALHL). In ears with ALHL, we have demonstrated the existence of EH (25), which might be responsible for the various categories of inner ear disease between ISSNHL and ALHL (26). Chen et al. (27) showed the existence of EH in patients with ISSNHL and vertigo on 3D-FLAIR after intratympanic injection of a GBCA, and speculated a causal relationship between EH and ISSNHL with vertigo; however, in patients with a history of ISSNHL in whom EH was detected, they considered that EH developed subsequent to ISSNHL (28).

Qin et al. (29) investigated the incidence of EH in affected ears based on four types of hearing loss and speculated that EH might be responsible for the pathology of the group with hearing loss at low frequencies, but that it was not related to prognosis. We investigated the existence of EH in ears with unilateral sensorineural hearing loss, including ISSNHL (30), and detected significant cochlear or vestibular EH in 24 and 14%, respectively, of the unaffected side of ears with ISSNHL. Another study conducted by our group regarding the existence of EH among patients with nonotological diseases found no significant vestibular EH in any patient (31).

## Discussion

Evaluation of inner ear disturbances in patients with ISSNHL has progressed since the application of 3D FLAIR, which enables pathophysiological elucidation of the disease. The fluid of the inner ear maintains its homeostasis by a variety of regulatory mechanisms, including an ion transport system, the BLB, and a constant blood supply (32). The BLB functions mainly in the stria vascularis and the spiral ligament in the cochlea. To summarize the reported 3D FLAIR findings, pre-contrast high signal may indicate minor hemorrhage or increased permeability of surrounding vessels to the perilymph, whereas post-contrast high signal indicates breakdown of the BLB. Moreover, hT_2_W-3D-FLAIR was reported to have higher sensitivity than regular 3D-FLAIR to signal alterations in the cochleae in ISSNHL (20). In our personal experience, no case of ISSNHL has exhibited pre-contrast low signal and post-contrast high signal on 3D-FLAIR; however, hT_2_W-3D-FLAIR revealed pre-contrast low signal and post-contrast high signal in 28.6% of cases (as shown in the Supplementary Figure S1).

In general, hearing recovery in ISSNHL is better at lower frequencies than at higher frequencies; however, some cases have a poor prognosis at low frequencies. Differences between ears with better or worse hearing recovery at lower frequencies may be related to the degree of inner ear disturbance among patients, although such differences have not been clinically elucidated. In our quantitative evaluation using hT_2_W-3D-FLAIR to identify lesion-specific disturbances, high post-contrast signal in the apical–middle turns was related to worse hearing recovery at low-tone frequencies, and low post-contrast signal in the apical–middle turns was related to better hearing recovery at low-tone frequencies (21). These results indicate irreversible changes with breakdown of BLB in the apical–middle turns that would lead to poor prognosis (Supplementary Figure S2). There is a difference in vascular permeability of the BLB between older and younger patients; i.e., greater vascular permeability of the BLB in older patients (23), and this difference may account for the poor prognosis in older patients with ISSNHL. Most cases of ISSNHL show unilateral hearing loss, and bilateral cases are rare. We applied hT_2_W-3D-FLAIR to evaluate the significance of high signal intensity in the endolymphatic duct (ED) (33). The ED is an insensitive component of the closed membranous labyrinth that acts as an endolymphatic channel, draining endolymph into the endolymphatic sac. High signal was observed bilaterally in a case of bilateral ISSNHL, suggesting an etiology of inner ear disturbance in cases of bilateral ISSNHL (33).

Detection of EH by MRI in ears with ISSNHL has provided new insights into the pathophysiology of the disease. Gürkov et al. have proposed a new terminology based on the symptomatic and imaging characteristics of various clinical entities, with the aim of clarifying and simplifying the diagnostic classification; i.e., primary hydropic ear disease and secondary hydropic ear disease (34, 35). The presence of EH in ears with ISSNHL is considered secondary EH (28).

In our investigation into the existence of EH in ears with definite MD and those with non-otological diseases, significant vestibular EH was present in 76.9% of affected ears but in none of the ears with non-otological diseases, and we concluded that EH in the vestibule was a useful indicator of definite MD (31). The percentage of significant vestibular EH in the unaffected side of ears with ISSNHL was 14% (30), which was higher than that in ears with non-otological diseases. The presence of EH in affected and unaffected ears in patients with unilateral ISSNHL suggests that (i) EH occurs secondary to the development of the disease, and (ii) pre-existing primary EH is a risk factor for the onset of ISSNHL (30).

**Figure 1:**
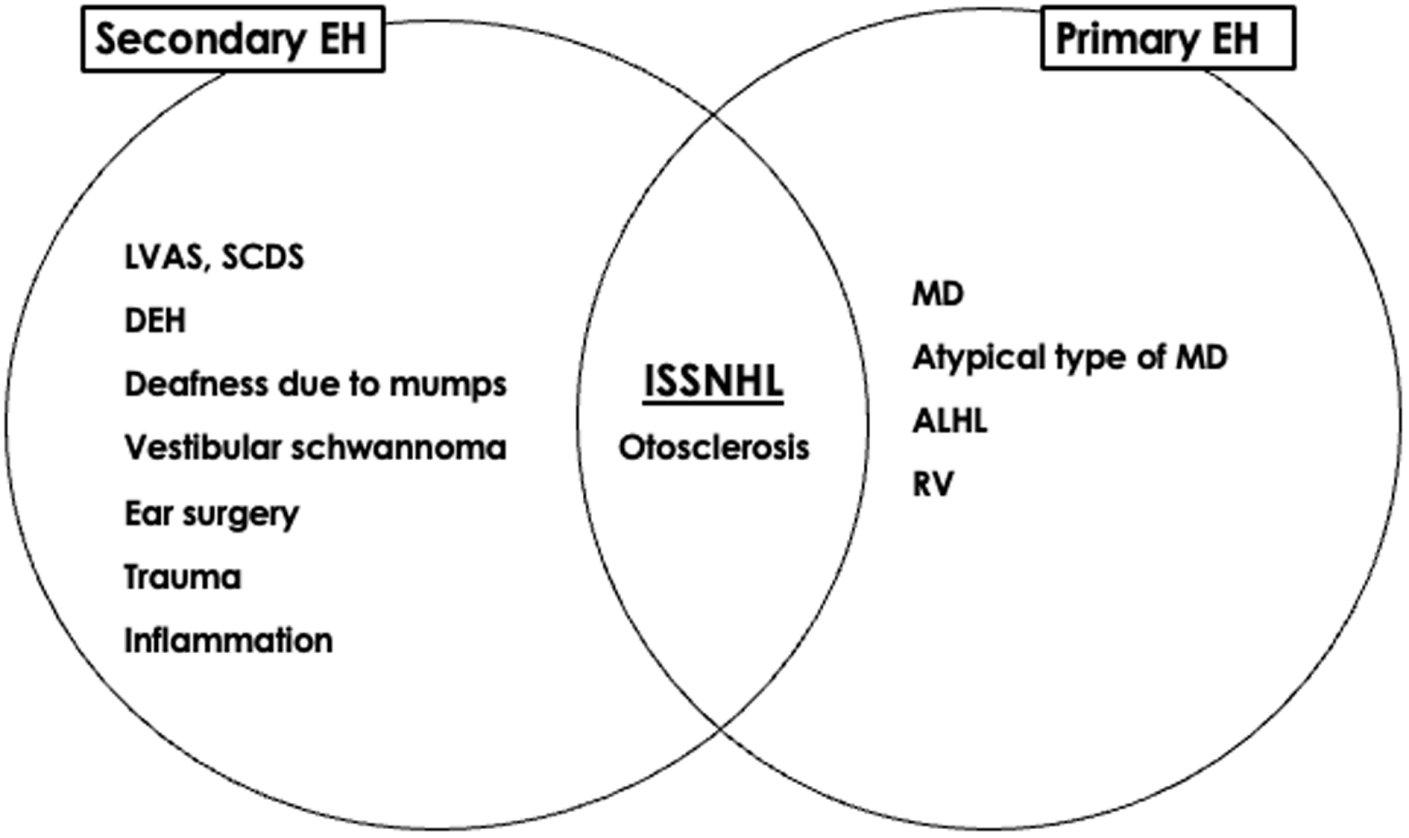
Significance of endolymphatic hydrops of idiopathic sudden sensorineural hearing loss from an etiological perspective [reprinted from reference (36)]. EH; endolymphatic hydrops, MD; Meniere’s disease, ALHL; acute low tone sensorineural hearing loss, RV; recurrent vestibulopathy, ISSNHL; idiopathic sudden sensorineural hearing loss, LVAS; large vestibular aqueduct syndrome, SCDS; superior canal dehiscence syndrome, DEH; delayed endolymphatic hydrops.

**Figure 2:**
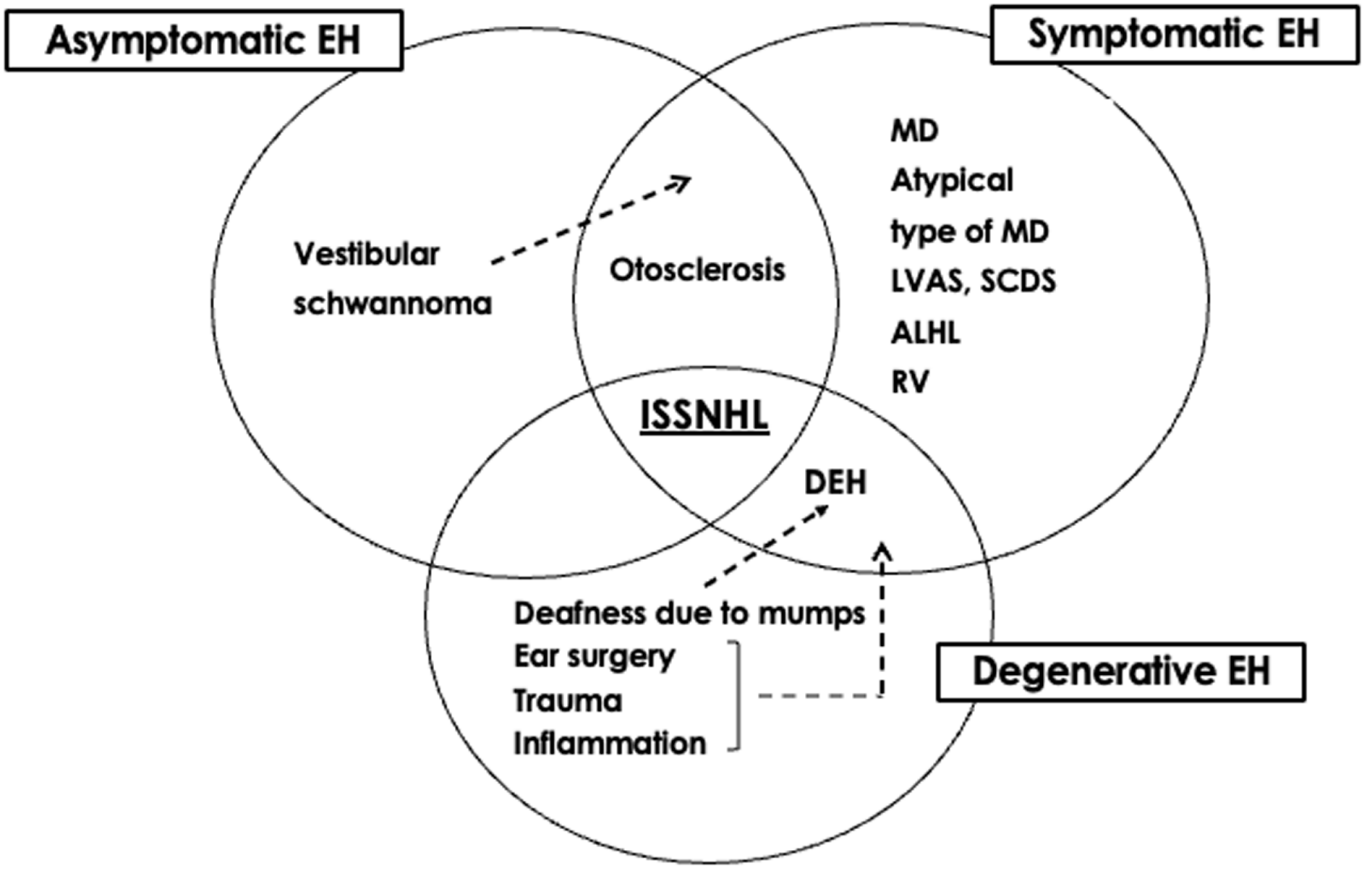
Significance of endolymphatic hydrops of idiopathic sudden sensorineural hearing loss from a therapeutic perspective [reprinted from reference (36)]. EH, endolymphatic hydrops; MD, Meniere’s disease; ALHL, acute low tone sensorineural hearing loss; RV, recurrent vestibulopathy; ISSNHL, idiopathic sudden sensorineural hearing loss; LVAS, large vestibular aqueduct syndrome; SCDS, superior canal dehiscence syndrome; DEH, delayed endolymphatic hydrops.

The significance of EH can be considered from etiological and therapeutic perspectives. The etiological classification of EH might help to summarize ideas for determining the pathophysiology of otological disorders, whereas the therapeutic classification provides clues to their management (36). From an etiological perspective, EH can be divided into primary and secondary forms. From a therapeutic perspective, EH can be divided into symptomatic, asymptomatic, and degenerative forms. The presence of EH in ears with ISSNHL might represent a combination of three possibilities: symptomatic EH is the primary etiology that requires treatment, asymptomatic EH is a risk factor requiring careful follow-up, and degenerative EH develops after acquiring the disease (Figures 1, 2). The mixed significance of EH might indicate various pathologies of the disease. Ears with ISSNHL that have significantly extended endolymphatic space may change to fluctuating sensorineural hearing loss (37).

Poor prognostic factors of ISSNHL include the severity of hearing loss, patient age, time from onset to treatment, and accompanying symptoms of vertigo/ dizziness or other diseases as complications. We should consider natural recovery of ISSNHL and limitations of existing evidence regarding the efficacy of medical interventions (1). The severity of inner ear disturbances among ears with ISSNHL varies, even when ears show similar levels of initial hearing loss. The present MRI evaluation can provide useful information for classifying the degree of inner ear disturbance in ears with ISSNHL.

## Conclusion

Analysis of ISSNHL using cutting-edge MRI analysis can provide useful information for elucidating its pathophysiology and predicting prognosis. Further investigations are necessary to enable the appropriate choice of treatment according to the pathophysiological findings.

## Author contributions

MS and SN contributed to writing the manuscript. MK and TY were involved in data analyses. All authors contributed to the article and approved the submitted version.

## Funding

This work was partially supported by Grants-in-Aid for Scientific Research from the Ministry of Education, Culture, Sports, Science and Technology of Japan (21K09605).

## Conflict of interest

The authors declare that the research was conducted in the absence of any commercial or financial relationships that could be construed as a potential conflict of interest.

## Publisher’s note

All claims expressed in this article are solely those of the authors and do not necessarily represent those of their affiliated organizations, or those of the publisher, the editors and the reviewers. Any product that may be evaluated in this article, or claim that may be made by its manufacturer, is not guaranteed or endorsed by the publisher.

## Supplementary material

The Supplementary material for this article can be found online at: https://www.frontiersin.org/articles/10.3389/fneur.2023.1193104/full#supplementary-material

## References

[1] Chandrasekhar SS Tsai Do BS Schwartz SR Bontempo LJ Faucett EA Finestone SA. Clinical practice guideline: sudden hearing loss (update). Otolaryngol Head Neck Surg. (2019) 161:S1–S45. doi: 10.1177/0194599819859885, PMID: 31369359

[2] Mark AS Seltzer S Nelson-Drake J Chapman JC Fitzgerald DC Gulya A. Labyrinthine enhancement on gadolinium-enhanced magnetic resonance imaging in sudden deafness and vertigo: correlation with audiologic and electronystagmographic studies. Ann Otol Rhinol Laryngol. (1992) 101:459–64. doi: 10.1177/000348949210100601, PMID: 1610062

[3] Mark AS Fitzgerald D. Segmental enhancement of the cochlea on contrast-enhanced MR: correlation with the frequency of hearing loss and possible sign of perilympatic fistula and autoimmune labyrinthitis. Am J Neuroradiol. (1993) 14:991–6. PMID: 8352175 PMC8333850

[4] Naganawa S Koshikawa T Nakamura T Kawai H Fukatsu H Ishigaki T. Comparison of flow artifacts between 2D-FLAIR and 3D-FLAIR sequences at 3 T. Eur Radiol. (2004) 14:1901–8. doi: 10.1007/s00330-004-2372-7, PMID: 15221269

[5] Sugiura M Naganawa S Teranishi M Nakashima T. Three-dimensional fluid-attenuated inversion recovery magnetic resonance imaging findings in patients with sudden sensorineural hearing loss. Laryngoscope. (2006) 116:1451–4. doi: 10.1097/MLG.0b013e318172ef85 16885752

[6] Nakashima T Naganawa S Sugiura M Teranishi M Sone M Hayashi H. Visualization of endolymphatic hydrops in patients with Meniere's disease. Laryngoscope. (2007) 117:415–20. doi: 10.1097/MLG.0b013e31802c300c 17279053

[7] Naganawa S Yamazaki M Kawai H Bokura K Sone M Nakashima T. Visualization of endolymphatic hydrops in Ménière’s disease with single-dose intravenous gadolinium- based contrast media using heavily T(2)-weighted 3D-FLAIR. Magn Reson Med Sci. (2010) 9:237–42. doi: 10.2463/mrms.9.237, PMID: 21187694

[8] Naganawa S Yamazaki M Kawai H BokuraK SM Nakashima T. Imaging of Meniere’s disease after intravenous administration of single-dose gadodiamide: utility of subtraction images with different inversion time. Magn Reson Med Sci. (2012) 11:213–9. doi: 10.2463/mrms.11.213 23037568

[9] Yoshida T Sugiura M Naganawa S Teranishi M Nakata S Nakashima T. Three-dimensional fluid-attenuated inversion recovery magnetic resonance imaging findings and prognosis in sudden sensorineural hearing loss. Laryngoscope. (2008) 118:1433–7. doi: 10.1097/MLG.0b013e318172ef85, PMID: 18475208

[10] Tagaya M Teranishi M Naganawa S Iwata T Yoshida T Otake H. 3 tesla magnetic resonance imaging obtained 4 hours after intravenous gadolinium injection in patients with sudden deafness. Acta Otolaryngol. (2010) 130:665–9. doi: 10.3109/00016480903384176, PMID: 19958242

[11] Berrettini S Seccia V Fortunato S Forli F Bruschini L Piaggi P. Analysis of the 3-dimensional fluid-attenuated inversion-recovery (3D-FLAIR) sequence in idiopathic sudden sensorineural hearing loss. JAMA Otolaryngol Head Neck Surg. (2013) 139:456–64. doi: 10.1001/jamaoto.2013.2659, PMID: 23681028

[12] Gao Z Chi FL. The clinical value of three-dimensional fluid-attenuated inversion recovery magnetic resonance imaging in patients with idiopathic sudden sensorineural hearing loss: a meta-analysis. Otol Neurotol. (2014) 35:1730–5. doi: 10.1097/MAO.0000000000000611, PMID: 25393973

[13] Lee JI Yoon RG Lee JH Park W Yoo MH Ahn JH. Prognostic value of labyrinthine 3D-FLAIR abnormalities in idiopathic sudden sensorineural hearing loss. Am J Neuroradiol. (2016) 37:2317–22. doi: 10.3174/ajnr.A4901, PMID: 27516239 PMC7963869

[14] Conte G Berardino FD Sina C Zanetti D Scola E Gavagna C. MR imaging in sudden Sensorineural hearing loss. Time Talk Am J Neuroradiol. (2017) 38:1475–9. doi: 10.3174/ajnr.A5230, PMID: 28546251 PMC7960407

[15] Song CI Pogson JM Andresen NS Ward BK. MRI with gadolinium as a measure of blood-labyrinth barrier integrity in patients with inner ear symptoms: a scoping review. Front Neurol. (2021) 12:662264. doi: 10.3389/fneur.2021.662264, PMID: 34093410 PMC8173087

[16] Liao WH Wu HM Wu HY Tu TY Shiao AS Castillo M. Revisiting the relationship of three-dimensional fluid attenuation inversion recovery imaging and hearing outcomes in adults with idiopathic unilateral sudden sensorineural hearing loss. Eur J Radiol. (2016) 85:2188–94. doi: 10.1016/j.ejrad.2016.10.005, PMID: 27842665

[17] Wang J Ren T Sun W Liang Q Wang W. Post-contrast 3D-FLAIR in idiopathic sudden sensorineural hearing loss. Eur Arch Otorhinolaryngol. (2019) 276:1291–9. doi: 10.1007/s00405-019-05285-z, PMID: 30747317

[18] Naganawa S Kawai H Sone M Nakashima T. Increased sensitivity to low concentration gadolinium contrast by optimized heavily T2-weighted 3D-FLAIR to visualize endolymphatic space. Magn Reson Med Sci. 9:73–80. doi: 10.2463/mrms.9.73 20585197

[19] Naganawa S Yamazaki M Kawai H Sone M Nakashima T. Contrast enhancement of the anterior eye segment and subarachnoid space: detection in the normal state by heavily T2-weighted 3D FLAIR. Magn Reson Med Sci. (2011) 10:193–9. doi: 10.2463/mrms.10.193, PMID: 21960002

[20] Naganawa S Kawai H Taoka T Suzuki K Iwano S Satake H. Heavily T2-weighted 3D-FLAIR improves the detection of cochlear lymph fluid signal abnormalities in patients with sudden sensorineural hearing loss. Magn Reson Med Sci. (2016) 15:203–11. doi: 10.2463/mrms.mp.2015-0065, PMID: 26597430 PMC5600057

[21] Yang CJ Yoshida YT Sugimoto S Teranishi M Kobayashi M Nishio N. Lesion-specific prognosis by magnetic resonance imaging in sudden sensorineural hearing loss. Acta Otolaryngol. (2021) 141:5–9. doi: 10.1080/00016489.2020.1827159, PMID: 33043763

[22] Pakdaman MN Ishiyama G Ishiyama A Peng KA Kim HJ Pope WB. Blood-labyrinth barrier permeability in Menière disease and idiopathic sudden sensorineural hearing loss: findings on delayed postcontrast 3D-FLAIR MRI. Am J Neuroradiol. (2016) 37:1903–8. doi: 10.3174/ajnr.A4822, PMID: 27256854 PMC7960486

[23] Yoshida T Kobayashi M Sugimoto S Teranishi M Naganawa S Sone M. Evaluation of the blood–perilymph barrier in ears with endolymphatic hydrops. Acta Otolaryngol. (2021) 141:736–41. doi: 10.1080/00016489.2021.1957500, PMID: 34346271

[24] Pyykkö I Nakashima T Yoshida T Zou J Naganawa. Ménière’s disease: a reappraisal supported by a variable latency of symptoms and the MRI visualisation of endolymphatic hydrops. BMJ Open. (2013) 3:e001555. doi: 10.1136/bmjopen-3502012-001555, PMID: 23418296 PMC3586172

[25] Shimono M Teranishi M Yoshida T Kato M Sano R Otake H. Endolymphatic hydrops revealed by magnetic resonance imaging in patients with acute low-tone sensorineural hearing loss. Otol Neurotol. (2013) 34:1241–6. doi: 10.1097/MAO.0b013e3182990e81, PMID: 23921924

[26] Yoshida T Sone M Kitoh R Nishio SY Ogawa K Kanzaki S. Idiopathic sudden sensorineural hearing loss and acute low-tone sensorineural hearing loss: a comparison of the results of a nationwide epidemiological survey in Japan. Acta Otolaryngol. (2017) 137:S38–43. doi: 10.1080/00016489.2017.1297539, PMID: 28366083

[27] Chen X Zhang XD Gu X Fang ZM Zhang R. Endolymphatic space imaging in idiopathic sudden sensorineural hearing loss with vertigo. Laryngoscope. (2012) 122:2265–8. doi: 10.1002/lary.23452, PMID: 22996668

[28] Ferster APO Cureoglu S Keskin N Paparella MM Isildak H. Secondary endolymphatic hydrops. Otol Neurotol. (2017) 38:774–9. doi: 10.1097/MAO.0000000000001377, PMID: 28306649 PMC5425947

[29] Qin H He B Wu H Li Y Chen J Wang W. Visualization of endolymphatic hydrops in patients with unilateral idiopathic sudden sensorineural hearing loss with four types according to chinese criterion. Front Surg. (2021) 8:682245. doi: 10.3389/fsurg.2021.682245, PMID: 34235173 PMC8255360

[30] Okazaki Y Yoshida T Sugimoto S Teranishi M Kato K Naganawa S. Significance of endolymphatic hydrops in ears with unilateral sensorineural hearing loss. Otol Neurotol. (2017) 38:1076–80. doi: 10.1097/MAO.0000000000001499, PMID: 28708796

[31] Yoshida T Sugimoto S Teranishi M Otake H Yamazaki M Naganawa S. Imaging of the endolymphatic space in patients with Ménière's disease. Auris Nasus Larynx. (2017) 45:33–8. doi: 10.1016/j.anl.2017.02.002 28256285

[32] Juhn SK Hunter BA Odland RM. Blood-labyrinth barrier and fluid dynamics of the inner ear. Int Tinnitus J. (2001) 7:72–83. PMID: 14689642

[33] Morimoto K Yoshida T Kobayashi M Sugimoto S Nishio N Teranishi M. Significance of high signal intensity in the endolymphatic duct on magnetic resonance imaging in ears with otological disorders. Acta Otolaryngol. (2020) 140:818–22. doi: 10.1080/00016489.2020.1781927, PMID: 32646259

[34] Gürkov R Pyykö I Zou J Kentala E. What is Menière’s disease? A contemporary re-evaluation of endolymphatic hydrops. J Neurol. (2016) 263:71–81. doi: 10.1007/s00415-015-7930-1, PMID: 27083887 PMC4833790

[35] Gürkov R. Menière and friends: imaging and classification of Hydropic ear disease. Otol Neurotol. (2017) 38:e539–44. doi: 10.1097/MAO.0000000000001479, PMID: 29135874

[36] Sone M Yoshida T Sugimoto S Kobayashi M Teranishi M Naganawa S. Pathological significance and classification of endolymphatic hydrops in otological disorders. Nagoya J Med Sci. (2022) 84:497–505. doi: 10.18999/nagjms.84.3.497, PMID: 36237884 PMC9529623

[37] Inui H Sakamoto T Ito T Kitahara T. Magnetic resonance imaging of endolymphatic space in patients with sensorineural hearing loss: comparison between fluctuating and idiopathic sudden sensorineural hearing loss. Acta Otolaryngol. (2020) 140:345–50. doi: 10.1080/00016489.2020.1720919, PMID: 32027202

