# Supplementary figures and images for: Pathophysiological analysis of idiopathic sudden sensorineural hearing loss by magnetic resonance imaging: A mini scoping review
Source: Front Neurol. 2023 Apr 20;14:1193104. doi: 10.3389/fneur.2023.1193104 (PMC10159174; doi:10.3389/fneur.2023.1193104)

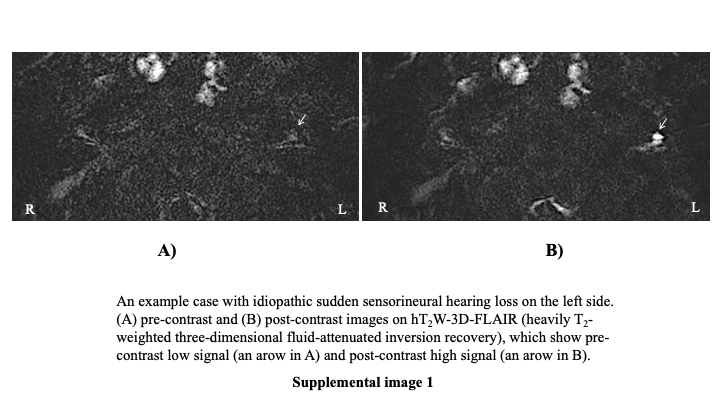

Supplement: Supplementary file 1 [file Image_1.TIFF]

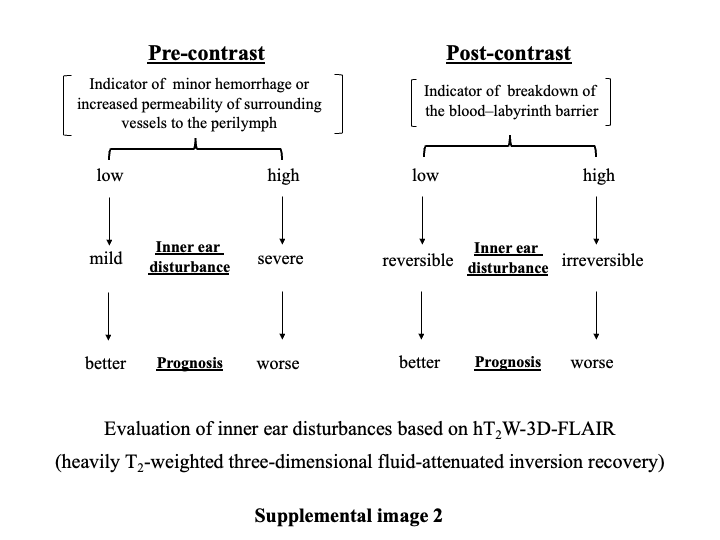

Supplement: Supplementary file 2 [file Image_2.TIFF]
